# Pigmented spindle cell variant of a thymic atypical carcinoid in an octogenarian

**DOI:** 10.1186/s40792-020-01094-3

**Published:** 2021-01-06

**Authors:** Yasushi Sakamaki, Ryo Tanaka, Daisuke Ishida, Hiromi Tsuji, Asako Mike, Hironao Yasuoka

**Affiliations:** 1 Department of Chest Surgery, Osaka Police Hospital, Kitayamacho 10-31, Tennoji, Osaka, 543-0035 Japan; 2 Department of Diagnostic Pathology, Osaka Police Hospital, Osaka, Japan

**Keywords:** Thymic neuroendocrine tumor, Thymic carcinoid, Pigmented carcinoid, Atypical carcinoid, Spindle cell carcinoid

## Abstract

**Background:**

A pigmented carcinoid is an extremely rare variant of carcinoid characterized by melanin pigmentation of the tumor, with only five cases described in the literature. In addition, thymic carcinoids are rare in elderly patients and their prognosis after resection of the carcinoid tumor is unclear.

**Case presentation:**

An anterior mediastinal tumor was incidentally found in an 82-year-old man who had been diagnosed with acute thoracic empyema. The tumor was considered most likely to be a noninvasive thymoma or thymic carcinoma for which surgery was indicated after the resolution of the empyema. The tumor was completely resected 4 months after the empyema surgery, and the patient had an uneventful postoperative course. A cut surface of the resected specimen was extensively pigmented and appeared dark-brownish, with abundant melanin pigmentation later confirmed in the spindle-shaped tumor cells. Based on the histologic examination and immunohistochemical study, melanoma was eliminated as a differential diagnosis and the tumor was diagnosed as a pigmented atypical carcinoid of the thymus.

**Conclusions:**

This report provides additional knowledge on thymic pigmented carcinoids and thymic atypical carcinoids in elderly patients.

## Background

A pigmented carcinoid (PC) is an extremely rare carcinoid variant. The unique histologic feature of PCs is that the tumor cells contain melanin pigment and show melanocytic differentiation. Herein, we report a case in which a thymic atypical carcinoid with melanin pigmentation was incidentally found as a solitary tumor of the anterior mediastinum in an octogenarian patient.

## Case presentation

**Fig. 1:**
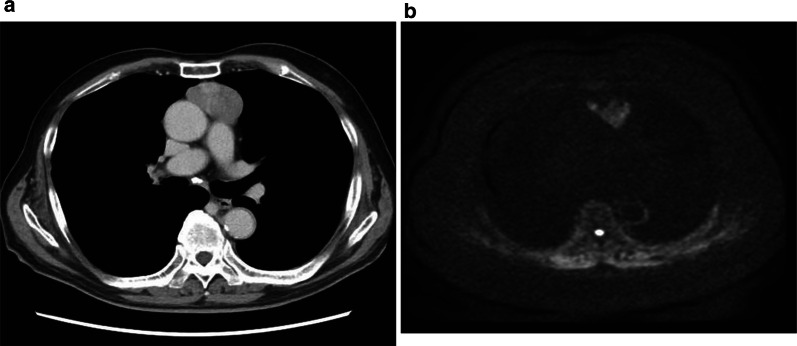
Tumor appearance on diagnostic imaging. **a** Computed tomography showing a solitary tumor in the anterior mediastinum. **b** Diffusion-weighted magnetic resonance image showing a high-intensity signal in the tumor

**Fig. 2:**
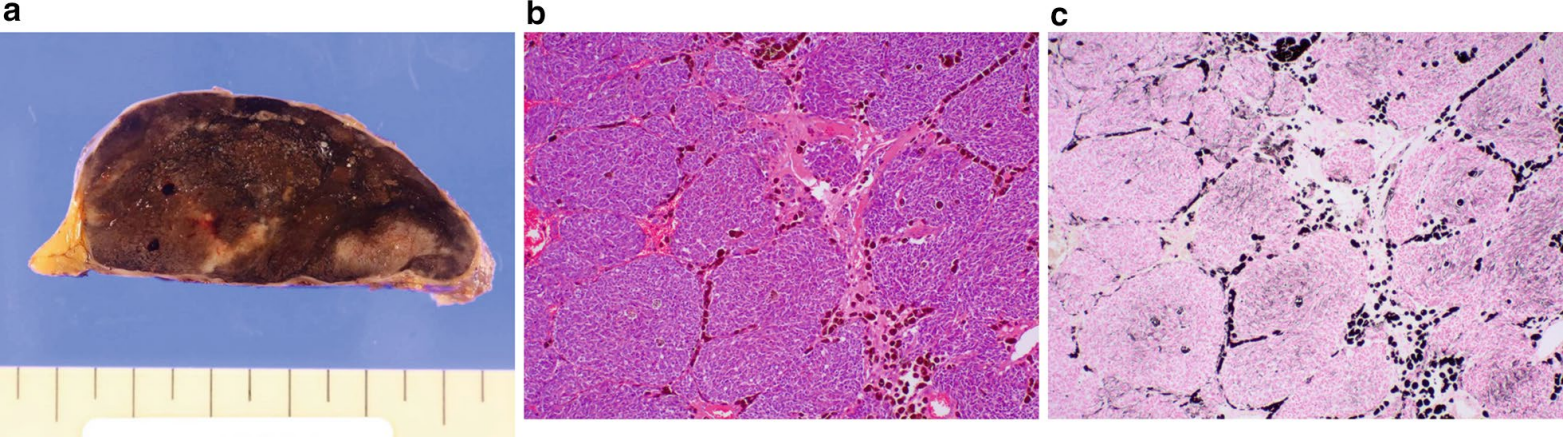
Gross appearance and histologic findings of the resected tumor. **a** Dark-brownish cut surface. **b** Hematoxylin–eosin staining revealing nesting proliferation of the spindle-shaped tumor cells and marked dark-brownish pigmentation in the macrophages. **c** Fontana–Masson staining confirming melanin in the pigmented cells

**Fig. 3:**
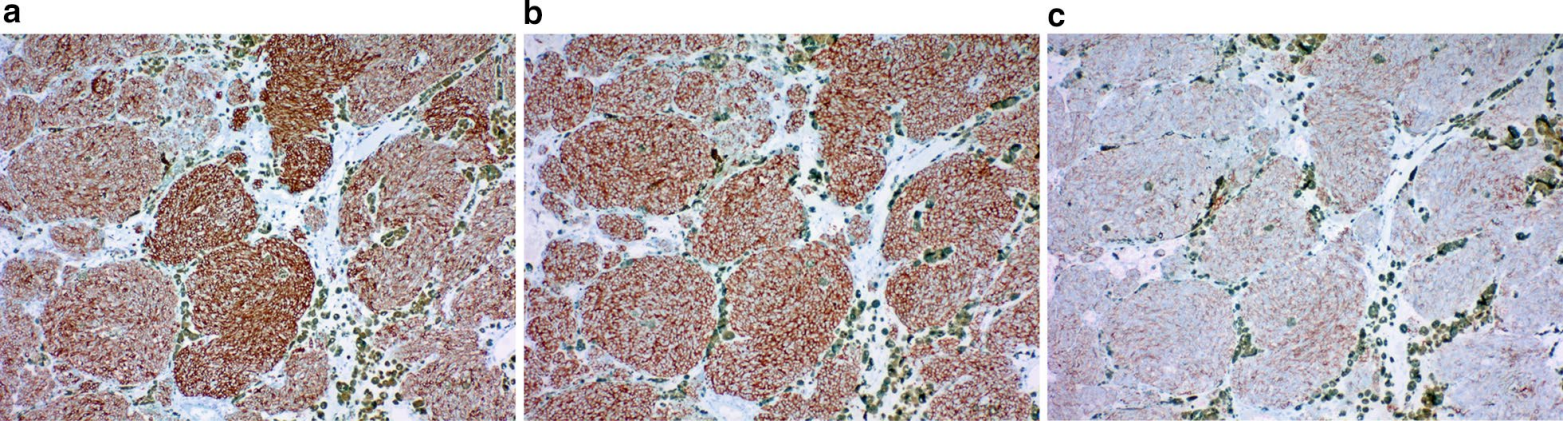
Immunoreactivity of the tumor cells revealing the epithelial and neuroendocrine nature. **a** Cytokeratin AE1/AE3. **b** Synaptophysin. **c** CD56

An 82-year-old man with no relevant medical history was admitted to our hospital with a diagnosis of acute left thoracic empyema. Computed tomography revealed a solitary mass measuring 7 × 5 × 3 cm in the anterior mediastinum (Fig. 1a). The computed tomography findings suggested no association between the mass and the empyema. The empyema resolved quickly after thoracoscopic surgery. The patient subsequently underwent further investigation of the mediastinal mass in accordance with the preoperatively provided informed consent. Magnetic resonance imaging revealed a high-intensity signal in a large part and a low-intensity signal in the remaining part in the mass on diffusion-weighted images, which confirmed that the lesion was a solid tumor with a liquid component, most likely a high-grade thymoma or thymic carcinoma (Fig. 1b). No other lesions were detected in the metastatic workup. At 4 months after the empyema surgery, thymectomy was performed through a median sternotomy. The tumor was completely resected and appeared well encapsulated, despite a small area of pericardial adhesion that was excised en bloc. A cut surface of the resected specimen appeared solid and dark-brownish, suggesting extensive pigmentation of the tumor components (Fig. 2a). Histologic examination revealed a well-circumscribed tumor constituted by organoid or nesting proliferation of spindle-shaped cells with necrosis (Fig. 2b). There were 2–4 mitotic figures per 10 high-power fields. Dark-brownish pigmentation was easily identifiable in the macrophages and dendritic cells but was less obvious in the tumor cells, reflecting the amount of melanin pigment contained in these cells, as confirmed by Fontana–Masson staining (Fig. 2c). Immunohistochemical study revealed the epithelial nature of the tumor cells based on positive reactivity with cytokeratins AE1/AE3 (Fig. 3a) and CAM5.2, but also revealed their neuroendocrine nature based on positive reactivity with synaptophysin and CD56 (Fig. 3b, c). In addition, there were numerous sustentacular cells surrounding the tumor cell nests, as confirmed by positivity for S-100. The Ki-67 labeling index was 18.7%. Despite positive reactivity of the tumor cells with vimentin, Melan-A, and HMB-45, melanoma was eliminated as a differential diagnosis based on positivity for the two above-mentioned cytokeratin markers and negativity for S-100. The tumor cells also showed negative reactions with epithelial membrane antigen, p63, p40, CD5, CD68, CD57, CD34, smooth muscle actin, CD99, leukocyte common antigen, glial fibrillary acidic protein, and carcinoembryonic antigen. Based on these findings and the opinions of external experts via personal communication, the tumor was finally diagnosed as a pigmented atypical carcinoid of thymic origin. The margins were clear and there was no invasion to the pericardium. However, the tumor had spread to the pericapsular fat tissue and one of the lymph nodes in the perithymic fat (T1aN1M0, stage IVa). Endocrinology laboratory tests exhibited normal values concerning the adrenal corticoid function. The patient has been doing well without adjuvant therapy. No signs of recurrence have been detected in the 9 months after surgery.

## Comments

Carcinoids are distinctive neuroendocrine tumors that occur most frequently in the gastrointestinal tract, followed by the respiratory tract [1]. Thymic carcinoids are uncommon tumors, accounting for 0.4–2% of all carcinoids and 2–4% of all anterior mediastinal tumors [1, 2]. Rosai and Higa first used the term ‘carcinoid tumor of the thymus’ to distinguish thymic carcinoids from thymomas based on their microscopic appearance [3]. Recent studies have clarified that carcinoids of the thymus are similar to those of bronchopulmonary origin, ranging in differentiation and behavior from a typical carcinoid to an atypical carcinoid (AC) to small-cell and large-cell carcinomas, despite the differences in genetic alterations between origins [4]. In accordance with the strategy for the classification of tumors in the lung, the World Health Organization currently defines typical carcinoid and AC of the thymus as low-grade and intermediate-grade neuroendocrine tumors, respectively [2].

A PC is an extremely rare variant of carcinoid that has often been reported to originate in the lung, although fewer than ten cases of bronchopulmonary origin have been described in the literature [5]. We found only five case reports of thymic PC to date in an online search of the English literature [6–9]. The reported cases of thymic PC are summarized in Table 1. The histogenesis of thymic PC is currently speculative [9]. Similar to patients with carcinoids, patients with thymic PC are predominantly male (80%) and young, ranging in age from 24 to 48 years (median, 46 years) [6–9]. Of these reported cases of thymic PC, two were functioning spindle cell carcinoids with ectopic secretion of adrenocorticotropic hormone [7, 9], while the other three were oval or round-to-oval cell carcinoids with no symptoms [6, 8]. The present case is the first case report of a nonfunctioning spindle cell PC of the thymus as well as that of a thymic PC in an elderly patient.

**Table 1:** Reported cases of pigmented carcinoid of the thymus

Case	Author	Age/sex	Symptom	Outcome	Size (mm)	Cell type	ACTH secretion	Mitosis	Necrosis
1	Ho and Ho [6]	46/M	None	ND	110	Oval	−	ND	+
2	Lagrange et al. [7]	48/F	EAS	ND	80	Spindle	+	Frequent	+
3	Klemm et al. [8]	32/M	None	NED for 10 years^a^	50^a^	Round to oval	−	3–10/10 HPF	+
4	Klemm et al. [8]	47/M	None						
5	Kuo [9]	24/M	EAS	Recurrence at 17 months postoperatively	60	Spindle	+	Rare	+
6	Present case	82/M	None	NED for 9 months	70	Spindle	−	2–4/10 HPF	+

This table is a revised version of a table in the report by Kuo [9]. Size represents the maximum diameter

*ACTH* adrenocorticotropic hormone, *ND* not described, *EAS* ectopic ACTH syndrome, *NED* no evidence of disease, *HPF* high-power field

^a^Outcome and size only reported for one unspecified case

The prognosis of our patient depends primarily on the aggressiveness of AC, given that melanin pigmentation has no definite prognostic implications [9]. Approximately 30% of ACs metastasize to organs such as the lung, liver, and bone [2]. Although our case has been indolent so far, close observation is needed because of the proven positive lymph node. As thymic carcinoid is a thymic neuroendocrine tumor (TNET), the prognosis of thymic carcinoid may be predicted based on recently reported treatment outcomes and prognostic factors for TNETs [10–13]. These studies reported promising outcomes after surgery, with 5-year overall survival rates ranging from 53 to 84.6%, and confirmed that complete resection is an important prognostic factor and is the optimal treatment for TNETs [10–13]. Concerning adjuvant therapy for TNETs, Filosso et al. [12] reported no statistical advantage in overall survival for adjuvant chemotherapy/radiotherapy based on an analysis of the largest series of TNETs studied to date.

## Conclusions

We treated an octogenarian patient with PC of thymic origin. PC is an extremely rare variant of carcinoids and this report provides additional knowledge on mediastinal PCs and thymic atypical carcinoids in elderly patients.

## Data Availability

The authors ensure that all the required datasets are presented in the main manuscript with no additional supporting files.

## Abbreviations

PC: Pigmented carcinoid
CD: Cluster of differentiation
HMB: Human melanoma black
AC: Atypical carcinoid
TNET: Thymic neuroendocrine tumor
